# A Clinically Relevant Drug Interaction Between Busulfan and Rifampin in the Setting of Hematopoietic Stem Cell Transplant Conditioning

**DOI:** 10.1002/prp2.70230

**Published:** 2026-03-13

**Authors:** Colleen Drangines, Janet Laquet, Ranju Kunwor

**Affiliations:** ^1^ Division of Hematology, Oncology, BMT, and Cellular Therapies SSM Health Saint Louis University Hospital; Saint Louis University School of Medicine St. Louis Missouri USA

**Keywords:** busulfan, HSCT, rifampin, therapeutic drug monitoring

## Abstract

Busulfan is an alkylating agent used in combination with other chemotherapeutic agents and has become a common component of conditioning regimens prior to hematopoietic stem cell transplantation. Busulfan has a very narrow therapeutic index corresponding to the area under the plasma concentration–time curve, with supra‐therapeutic busulfan levels associated with hepatic and neurologic toxicity and increased transplant‐related mortality, while sub‐therapeutic levels can be ineffective, resulting in disease relapse or graft failure. Busulfan is believed to be metabolized in the liver via conjugation with glutathione as well as cytochrome P450 isoenzymes. Interactions with medications known to affect CYP3A4, including phenytoin and metronidazole, have been described in multiple instances. This case discusses a drug–drug interaction between busulfan and rifampin, a known CYP3A4 inducer that has limited evidence currently in the literature. Busulfan therapeutic drug monitoring revealed accelerated busulfan clearance determined to be due to rifampin's effect on busulfan metabolism.

## 1 Introduction

Busulfan (1,4‐butanediol dimethanesulfonate) is an alkylating agent that has been used as part of conditioning regimens prior to hematopoietic stem cell transplantation (HSCT) for over 20 years [1, 2]. Similar to other therapies based on DNA alkylation, busulfan has a very narrow therapeutic index (NTI). Busulfan is a known NTI drug as defined by the US FDA. For all NTI drugs, a small change in the dose or blood concentration of these NTI drugs may lead to therapeutic failure or serious adverse events. NTI is determined based on population‐level pharmacokinetic (PK)/pharmacodynamic (PD) and/or exposure/dose response data, and exposure–response curve within individual or patient‐level data. Within‐subject variability of NTI drugs is estimated with bioequivalence tests such as Clearance (C max) and AUC_0‐t_ [3]. Today, an intravenous busulfan‐ and fludarabine‐based regimen has been adopted at many transplant facilities as the standard‐of‐care condition regimen for myeloid diseases, with available clinical results indicating it is generally safe and effective [1]. While the development of a busulfan intravenous formulation greatly reduced the inter‐individual variability in PK and bioavailability seen with its oral formulation, clinically significant variation between patients continues to be noted [1, 4]. Supra‐therapeutic exposure of busulfan has been associated with BMT treatment‐related toxicities, including sinusoidal obstruction syndrome (SOS), neurological disturbances, and increased transplant‐related mortality. Sub‐therapeutic levels of busulfan are associated with insufficient efficacy that can lead to disease relapse and graft failure [1, 2, 4, 5, 6, 7]. Therefore, it is standard to monitor therapeutic index or PK laboratories of busulfan for each patient receiving myeloablative dose busulfan.

Busulfan's NTI makes correctly dosing and adjusting for possible drug–drug interactions (DDIs) critical for patient outcomes. Despite being used for decades, the mechanisms of busulfan PK‐based DDIs and potential downstream effects of these interactions have not been fully characterized [4, 7]. This is in part because busulfan's global metabolic profile remains unsettled. Busulfan metabolism is best described by hepatic glutathione conjugation via glutathione‐S‐transferase (GST) isoenzymes, although additional indirect evidence from pharmacogenomic studies has indicated that other oxidative enzymes, including those of the cytochrome P450 family, likely contribute to the drug's clearance [1, 2, 7]. There have been multiple reports of DDIs of busulfan with medications known to affect the CYP3A4 system and its metabolites (e.g., phenytoin, itraconazole, and metronidazole) [2, 4, 5, 6, 7, 8, 9]. In 2005, a possible interaction between busulfan and rifampin, a medication used for tuberculosis that is a known CYP3A4 inducer, was reported in the case of a 3‐year‐old female [10]. However, there has since been no additional studies or cases of this interaction published in the literature. In this case report, we describe an interaction between busulfan and rifampin in an adult that provides further evidence of a clinically significant drug–drug interaction.

## 2 Case

The patient is a 61‐year‐old female with a past medical history of hypothyroidism, dyslipidemia, gastroesophageal reflux disease, and triple negative breast cancer s/p bilateral mastectomy who was diagnosed in November 2023 with Acute Myeloid Leukemia (AML) with monocytic differentiation and + MLL rearrangement, an adverse risk AML due to translocation (t(v;11q23.3)/KMT2A‐rearranged). Lumbar puncture did not indicate CNS involvement. Induction therapy with cytarabine and idarubicin (7 + 3) was administered. After achieving remission, consolidation therapy of high‐dose cytarabine was given for three cycles while awaiting matched unrelated donor (MUD) Allogeneic HSCT consolidation. Bone marrow biopsy confirmed the patient was in complete remission, MRD negative, prior to transplant. Significant pre‐transplant testing results included a positive quantiferon for tuberculosis. A CT scan of the chest confirmed the patient did not have active tuberculosis. Infectious Disease was consulted, with a recommendation to initiate rifampin 600 mg by mouth daily for tuberculosis prophylaxis.

**FIGURE 1:**
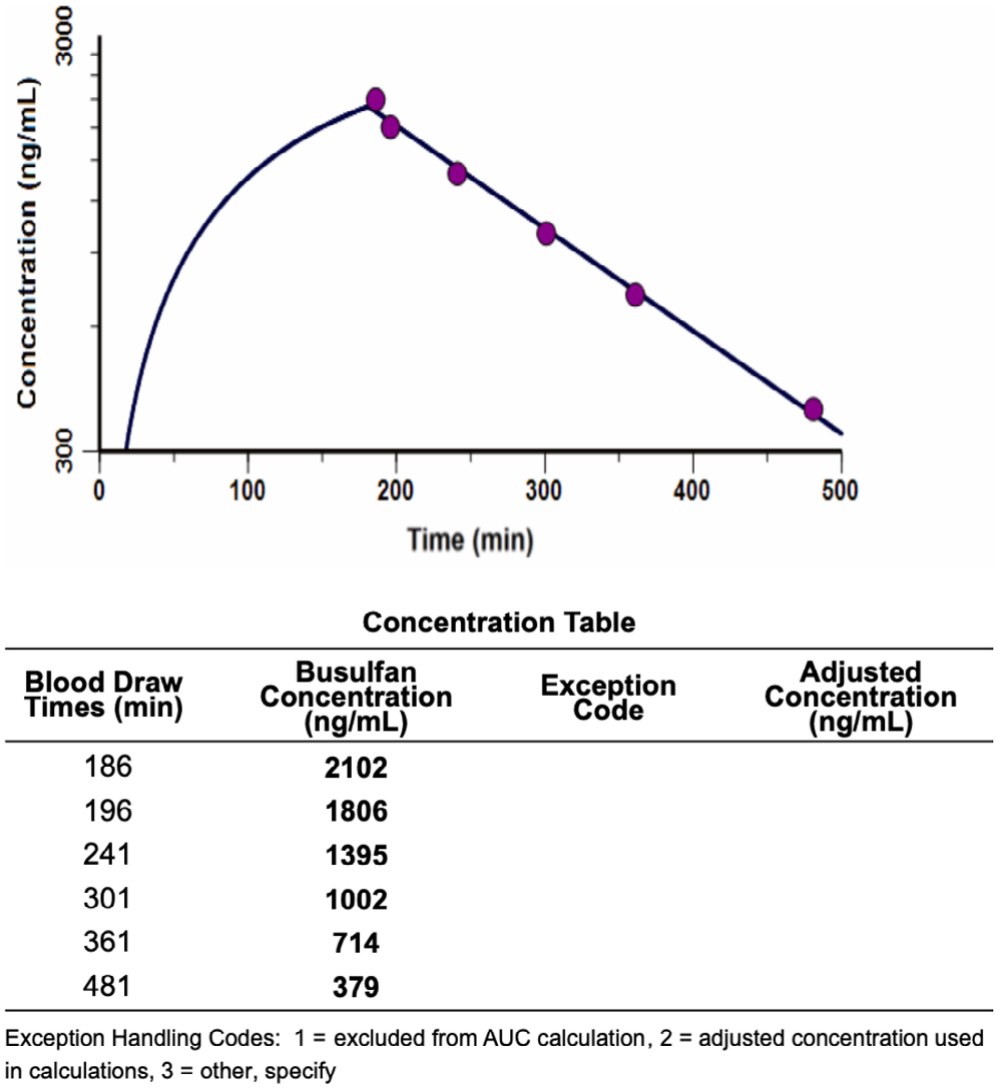
Clearance of Busulfan Dose #1.

The patient proceeded to a myeloablative, MUD Allo HSCT using a conditioning regimen of fractionated busulfan, fludarabine, and post‐transplant cyclophosphamide, according to institutional protocol [11]. The weight used for dose calculations was 85.6 kg (ideal body weight). On Day −20, an initial dose of 150.60 mg (1.76 mg/kg/dose) of busulfan was given, with a cumulative harmonized AUC target range of 72.2 to 92.0 mg × h/L over 6 total doses. Both infusion and sampling were reported to occur without issues. PK laboratories were performed with Dose #1 and Dose #3 for therapeutic drug monitoring purposes; assays were performed with stringent quality controls. Dose #1 showed an observed busulfan clearance of 3.00 mL/min/kg, approximately average for the patient's age and dosing body weight (Figure 1). However, the observed elimination half‐life, 122 min, was more consistent with a clearance closer to 1 standard deviation faster (average half‐life in adults is 162 + − 44 min). The harmonized AUC exposure from Dose #1 was lower than expected, found to be 9.51 mg × h/L. Given the lack of other significant potential DDIs, rifampin, a known potent CYP3A4 inducer, was suspected to be the cause of the reduced exposure seen with Dose #1.

**FIGURE 2:**
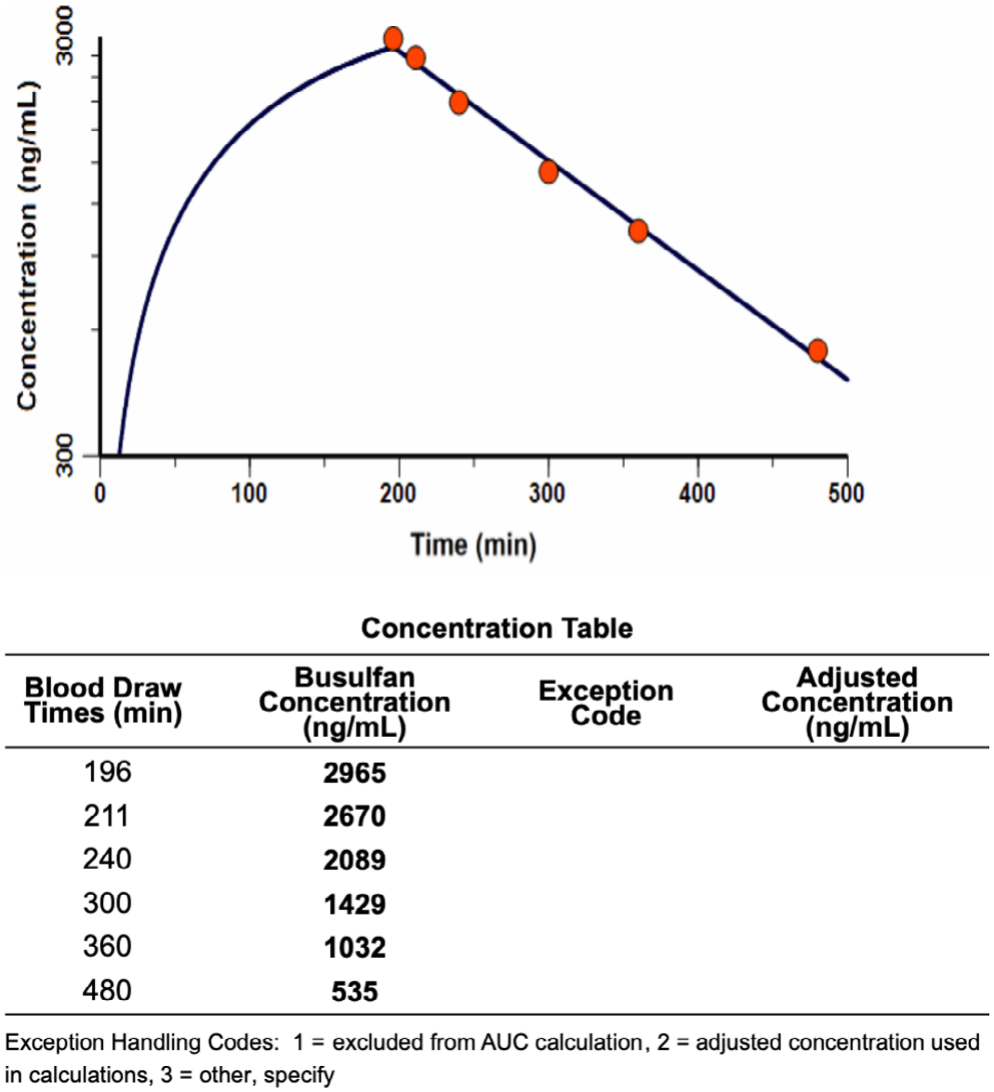
Clearance of Busulfan Dose #3.

Consequently, a 219.0 mg (2.56 mg/kg/dose) dose, a 45.4% increase from Dose #1, was administered for Doses #3 and #4. This was estimated to provide individual dose exposures (AUCs) of 13.83 mg × h/L to result in an estimated cumulative exposure of 74.4 mg × h/L over the planned 6‐dose course. Follow‐up PK analysis performed with Dose #3 showed an observed clearance of 3.11 mL/min/kg, harmonized AUC exposure of 13.37 mg × h/L, and elimination half‐life of 115 min (Figure 2). Both the clearance and half‐life of Dose #3 were consistent with those of Dose #1 (within 4% and 6%, respectively). Notably, Dose #3 was administered with fludarabine per the conditioning protocol; concomitant fludarabine administration has been observed to decrease busulfan clearance by an average of 10%–15% over the first 3 days of conditioning, suggesting an even greater impact by Rifampin [12]. Based on these results, Dose #5 of busulfan was further increased to 240 mg (2.80 mg/kg/dose) to provide an estimated individual dose AUC of 14.90 mg × h/L and estimated cumulative AUC of 75.8 mg × h/L. PK laboratories from Dose #4 revealed similar trends, leading to an increase to 249 mg (2.91 mg/kg/dose) of busulfan for the sixth and final dose (Table 1).

**TABLE 1:** Myeloablative Conditioning Protocol Followed by Matched Unrelated Hematopoietic Progenitor Cell Transplant [13].

Day	Drug	Dose
−20	Busulfan	80 mg/m2 IV
−13	Busulfan	80 mg/m2 IV
−6	Busulfan Fludarabine	Dose per PK on Day −20 IV 40 mg/m2 IV
−5	Busulfan Fludarabine	Dose per PK from Day −20 IV 40 mg/m2 IV
−4	Busulfan Fludarabine	Dose per PK from Day—6 IV 40 mg/m2 IV
−3	Busulfan Fludarabine	Dose per PK from Day—5 IV 40 mg/m2 IV
−2	Rest	
−1	Rest	
0	HPC infusion	
+ 3	Cyclophosphamide Mesna	50 mg/kg 50 mg/kg
+4	Cyclophosphamide Mesna 50 mg/kg	50 mg/kg 50 mg/kg
+5	Tacrolimus Mycophenolate	0.045 mg/kg PO BID 15 mg/kg PO TID (max dose 1000 mg PO TID)
+7	Filgrastim	480 mcg SQ daily until ANC > 1500 × 3 days

## 3 Discussion

This case featured a significant increase in busulfan dosing, indicated by PK laboratory results that revealed an increased clearance and consequently decreased exposure of the drug. There are no prior reports or studies in the current literature that discuss potential DDIs between Rifampin and busulfan. AUC as seen in the PK laboratory results, and the lack of any other potential interaction suggests the Rifampin as the main stakeholder in this DDI. Rifampin is a known inducer of CYP3A4, the isoform within the CYP450 superfamily most often associated with severe drug interactions [2, 4]. Phenytoin, another CYP3A inducer, is believed to affect metabolism of busulfan. Case reports have indicated that combined busulfan and phenytoin use results in lower than predicted busulfan levels [2, 4, 7, 9]. Furthermore, it has been reported that concomitant administration with metronidazole leads to significantly increased busulfan levels and a corresponding increased prevalence of SOS [2, 4, 6, 7, 8, 9]. These findings are suspected to occur, at least in part, due to competition between the two medications for CYP3A4 oxidation. Itraconazole has also been widely reported to lead to reduced busulfan clearance [2, 4, 5, 6, 9]. A recent case reported indicated that use of blinatumomab, a protein therapeutic that elevates cytokine levels, concurrently with busulfan was associated with significantly increased busulfan levels [9]. While the precise role of CYPs in the metabolism of busulfan has not been determined, this indirect evidence, as well as the fact that busulfan's metabolites (THT and 3‐hydroxysulfolane) can theoretically be substrates for CYP450 enzymes, suggests CYP3A4 is involved in some capacity [4]. The increased observed clearance of busulfan in this case in the setting of concomitant Rifampin use, and the absence of other significant DDIs, both supports this DDI. Our case introduces relevant consideration for clinicians utilizing busulfan in HSCT conditioning regimens and concomitant Rifampin use. This case additionally exemplifies the importance of therapeutic drug monitoring throughout treatment with busulfan to allow for appropriate dose adjustments and risk reduction.

The HSCT patient population has been found to be at an increased risk for DDIs as, on average, these patients take more medications and are often exposed for long periods of time to complex drug regimens [4, 6, 7, 14]. As systemic exposure to busulfan outside its narrow therapeutic window has been linked to an increased incidence of complications and poorer outcomes, it is important that further reports, discussions, and investigations of DDIs involving busulfan are prioritized among oncology providers and researchers. Dose adjustments as per therapeutic monitoring are essential to avoid serious therapeutic failure due to subtherapeutic dose. Today's routine use of busulfan in pre‐transplantation conditioning regimens means that such DDIs have the potential to affect a significant number of patients. Furthermore, this at‐risk population is likely to continue to grow, as busulfan's relative success within HSCT conditioning for myeloid malignancies has prompted its use in developing and improving other preparative regimens [1]. In terms of the outcome of our case, the patient had an appropriate response to busulfan as evidenced by myeloablation/nadir blood count based on post transplant CBCs, and more importantly, she had the expected course of count recovery with no serious adverse events within 100 days and 1 year post transplant. Our patient is now more than a year post transplant, has been disease free, with normal pulmonary function tests at one year post transplant evaluation, has excellent quality of life, and is working full time.

Treosulfan is a novel prodrug of a bifunctional alkylating agent, which does not require enzymatic activation or hepatic metabolism. It has a low inter‐ and intrapatient variability and does not require drug‐level monitoring and adjustments. Treosulfan is approved by the FDA in combination with Fludarabine as a HSCT conditioning regimen [15]. Treosulfan has shown to be non‐inferior to busulfan in clinical trials [16, 17, 18, 19]. Our case also highlights the consideration of using an alternative agent when possible to avoid DDI in select patients.

## 4 Conclusion

While busulfan is a key component of many HSCT conditioning regimens, it is also reported to be involved in many drug–drug interactions. This case presents evidence of a clinically significant interaction between busulfan and rifampin that has only been reported once before in the literature. Previous reports of DDIs involving busulfan strongly support that CYP3A4 plays a role in the drug's metabolism, which is in alignment with the mechanism reported in this case. Given busulfan's narrow therapeutic window and the well‐documented adverse effects associated with supra‐therapeutic levels and serious therapeutic failure with subtherapeutic levels, continued awareness and discussion about DDIs that can alter busulfan's clearance are crucial to reducing risks when treating HSCT patients. Our case highlights the importance of strict therapeutic drug monitoring of NTI drugs, and clinicians may even explore the use of alternative HSCT conditioning regimens in patients who are susceptible to DDI.

## Author Contributions

C.D. performed literature review and drafted the manuscript. R.K. conceived of the case report, collected clinical data, and supervised the project. J.L. assisted with analysis and interpretation.

## Funding

The authors have nothing to report.

## Disclosure

The authors confirm that the principal investigator for this paper is Dr. Kunwor and that she had direct clinical responsibility for patients.

## Ethics Statement

SLU IRB has determined that a descriptive report of observations on up to five people or organization(s), in which the observations were retrieved in a retrospective manner and no research questions/hypotheses are being tested, does not meet the definition of research, and may be considered a ‘case report’. In such cases, SLU investigators are not required to obtain IRB approval prior to beginning the activity. However, these investigators have made reasonable attempts to protect the patient's/individual's privacy by removing identifiers.

## Conflicts of Interest

The authors declare no conflicts of interest.

## Data Availability

The data underlying this case report contain personal health information and cannot be shared publicly to protect patient privacy. De‐identified data may be available from the corresponding author upon reasonable request and with appropriate ethics approvals.
